# Supplementary material for: Learning curve and surgical outcome of robotic assisted colorectal surgery with ERAS program
Source: Sci Rep. 2022 Nov 29;12:20566. doi: 10.1038/s41598-022-24665-w (PMC9709162; doi:10.1038/s41598-022-24665-w)
Supplement: Supplementary file 2 — Supplementary Information 2. [file 41598_2022_24665_MOESM2_ESM.zip › RAL Raw data-Ñ[▒K/084/Patho_ñw╝╨░O▒Kñσ.pdf]

臺中榮民總醫院 臺中市臺灣大道四段1650號 病理部活體組織切片 臨床醫學實驗及病理診斷中心 檢驗(查)報告

[redacted]

Pathologic diagnosis:
1. Rectum, robotic low anterior resection --- Status post polypectomy at other hospital with ulcer and scar formation. No residual tumor seen. The bilateral cut ends and perirectal lymph nodes (0/15) are free of tumor.
2. IMA root, dissection --- Unremarkable fibroadipose tissue and lymph nodes (0/6).
3. Rectum, cut end, distal, resection --- Negative for malignancy.

Ancillary study for diagnosis:
Recut and AE1/AE3 (no abnormal epithelial pattern) IHC stain done for section C.

Prognostic and predictive factor:
1. Tumor size: Not applicable.
2. Depth of invasion: No evidence of primary tumor.
3. Histologic grade: Not applicable.
4. Angiolymphatic invasion: Not identified.
5. Perineural invasion: Not identified.
6. Tumor deposits (discontinuous extramural extension): Not identified.
7. Macroscopic tumor perforation: Not applicable.
8. Surgical Margins: a) Proximal Margin: Uninvolved by invasive carcinoma. b) Distal Margin: Uninvolved by invasive carcinoma. c) Radial or Mesenteric Margin: Uninvolved by invasive carcinoma.
9. Lymph node status: Uninvolved (0/21).
10. Extranodal involvement: Not identified.
11. Tumor regression grading S/P CCRT: No known presurgical therapy.
12. Type of polyp in which invasive carcinoma arose: Not applicable.
13. Additional pathologic findings: Not identified.
14. Pathological TNM Stage: pTxNx(According to the eighth edition, American Joint Committee on Cancer Staging Guidelines for Tumors).
15. TNM descriptors: Not applicable.

Gross description:
The specimen consists of 1) a segment of colon, 14 cm in length and 4 cm in proximal cut end circumference, 5.5 cm in distal circumference, and perirectal soft tissue. A scar-like lesion with focal ulceration marked by tatto, 1x1 cm, is noted, 1.5 cm to the distal cut end. On cross section, there is no mass lesion seen grossly. The both cut ends are free of tumor grossly. 2) IMA root, a piece of tan yellow soft tissue, 3.5x2.5x0.8 cm. 3) distal cut end, a piece of tan mucosa-covered soft tissue, 2x1.8x1.2 cm. Representative part for section: A) proximal cut end B) distal cut end C-E) all of lesion F) all of specimen 2 G) specimen 3 H-S) lymph nodes. Blue ink indicated serosa; yellow ink indicated radial margin.
#T-59600_2 #M-38000_2 0 1105 SP0000
#T-C4560_2 #M-09183_2 0 1105 000000

已電子簽章

警告：本單僅供醫師參考使用，不具任何申請效力！！
請病患妥為保管，若不慎遺失需自負個人資料外洩責任！
